# Supplementary material for: Patients with knee osteoarthritis have altered gait and gaze patterns compared to age-matched controls: A pilot study
Source: PLoS One. 2023 Nov 27;18(11):e0283451. doi: 10.1371/journal.pone.0283451 (PMC10681189; doi:10.1371/journal.pone.0283451)
Supplement: S1 Fig — Dynamometer is placed at the posterior distal calf with the stabilizing strap. (DOCX) [file pone.0283451.s001.docx]

Appendix


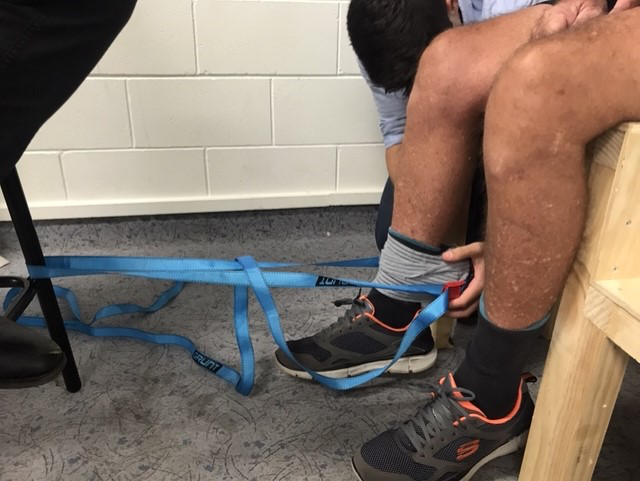


Figure 1: Demonstration of isometric maximal force contraction for knee flexion. Dynamometer is placed at the posterior distal calf with the stabilizing strap.
