# Supplementary material for: Patients with knee osteoarthritis have altered gait and gaze patterns compared to age-matched controls: A pilot study
Source: PLoS One. 2023 Nov 27;18(11):e0283451. doi: 10.1371/journal.pone.0283451 (PMC10681189; doi:10.1371/journal.pone.0283451)
Supplement: Attachment — Submitted filename: Feedback from Editors + repsonse.docx [file pone.0283451.s002.docx]

Reviewers comments and authors responses

Reviewer’s comment:

1. Please ensure that your manuscript meets PLOS ONE's style requirements, including those for file naming. The PLOS ONE style templates can be found at 
<https://journals.plos.org/plosone/s/file?id=wjVg/PLOSOne_formatting_sample_main_body.pdf> and 
<https://journals.plos.org/plosone/s/file?id=ba62/PLOSOne_formatting_sample_title_authors_affiliations.pdf>

Author’s response: Thank you for sending through the links for formatting style. The author/s have reviewed the formatting templates and modified the manuscripts accordingly.

Reviewer’s comment:

2. PLOS requires an ORCID iD for the corresponding author in Editorial Manager on papers submitted after December 6th, 2016. Please ensure that you have an ORCID iD and that it is validated in Editorial Manager. To do this, go to ‘Update my Information’ (in the upper left-hand corner of the main menu), and click on the Fetch/Validate link next to the ORCID field. This will take you to the ORCID site and allow you to create a new iD or authenticate a pre-existing iD in Editorial Manager. Please see the following video for instructions on linking an ORCID iD to your Editorial Manager account: <https://www.youtube.com/watch?v=_xcclfuvtxQ>

Author’s response: The Authors ORCID iD is 0000-0002-8324-6801

Reviewer’s comment:

3. Please amend the manuscript submission data (via Edit Submission) to include author  Kaushik Hazratwala.

Author’s response: The author will be sure to add the above author in the manuscript submission.

Reviewer’s comment:

4. We note you have included a table to which you do not refer in the text of your manuscript. Please ensure that you refer to Table 5 in your text; if accepted, production will need this reference to link the reader to the Table.

Author’s response: Thank you for notifying the author of this. Table 5 is now referred to within the text.

Reviewer's Responses to Questions

**Comments to the Author**

1. Is the manuscript technically sound, and do the data support the conclusions?

The manuscript must describe a technically sound piece of scientific research with data that supports the conclusions. Experiments must have been conducted rigorously, with appropriate controls, replication, and sample sizes. The conclusions must be drawn appropriately based on the data presented.

Reviewer #1: Partly

Reviewer #2: Partly

2. Has the statistical analysis been performed appropriately and rigorously?

Reviewer #1: No

Reviewer #2: I Don't Know

3. Have the authors made all data underlying the findings in their manuscript fully available?

The [PLOS Data policy](http://www.plosone.org/static/policies.action#sharing) requires authors to make all data underlying the findings described in their manuscript fully available without restriction, with rare exception (please refer to the Data Availability Statement in the manuscript PDF file). The data should be provided as part of the manuscript or its supporting information, or deposited to a public repository. For example, in addition to summary statistics, the data points behind means, medians and variance measures should be available. If there are restrictions on publicly sharing data—e.g. participant privacy or use of data from a third party—those must be specified.

Reviewer #1: Yes

Reviewer #2: Yes

4. Is the manuscript presented in an intelligible fashion and written in standard English?

PLOS ONE does not copyedit accepted manuscripts, so the language in submitted articles must be clear, correct, and unambiguous. Any typographical or grammatical errors should be corrected at revision, so please note any specific errors here.

Reviewer #1: Yes

Reviewer #2: No

5. Review Comments to the Author

Please use the space provided to explain your answers to the questions above. You may also include additional comments for the author, including concerns about dual publication, research ethics, or publication ethics. (Please upload your review as an attachment if it exceeds 20,000 characters)

Reviewer #1: I want to thank the authors for their important work. This topic is of great interest, and is close to my heart. I can tell the design, performance, and analysis of the study, along with the preparation of this manuscript, took a lot of time and effort. Thank you for the opportunity to review this manuscript.

Reviewer’s comment:
Overall, I think it's important to remember the authors present this as a pilot study, which is something I think gets lost when communicating the findings of this current investigation. This is especially true in the way the discussion and conclusions are presented. I would assume this study will be followed up with a larger, appropriately powered investigation, hence the absence of an a priori analysis for this study. If the methods and sample size are designed for a pilot, then the discussion and conclusions should be more aimed at discussing and making conclusions aimed at preparing for the next study, and not on trying to make definitive statements.

Author’s response: Thank you for acknowledging the time taken to produce this manuscript. The discussion and conclusion have been reworded to be more in line with being a pilot study.

Reviewer’s comment: I think a quick proofread for grammatical errors is appropriate, (e.g., lines 15 "behavior" should be "behaviors", 20 remove "more", 135 "climba" should be "climb", etc.).

Author’s response: The manuscript has now been proof read and the above errors corrected.

INTRODUCTION
Refs 12, 13, 14 are appropriate given the topic, but there are some newer publications that would also be beneficial to include.

Reviewer’s comment: The authors do not state the alternate hypothesis in the INTRODUCTION, and only state it formally in the DISCUSSION (line 219-220).

Author’s response: The alternate hypothesis has now been removed from the manuscript as this is a pilot study and therefore no hypothesis is required.

METHODS
Reviewer’s comment: Lines 74-79 which describe descriptive data of the participants is really more appropriate in the RESULTS section.

Author’s response: The lines above have been moved to the results section.

Reviewer’s comment: Controls were "apparently healthy." Can you operationally define this?

Author’s response: “Apparently healthy” has been removed from the manuscript due to being unable to operationally define this term.

Reviewer’s comment: Experimental group were those with "severe KOA." What severity grading was used? Kellgren-Lawrence? This needs to be stated and included.

Author’s response: Thank you for this suggestion. The Kellgren-Lawrence was utilized and has been stated and included in the manuscript.

Reviewer’s comment: How was age matching performed (e.g., exact age or +/- a certain number of years)? Was sex matching performed as well? The authors state in the results there was no difference in sex overall. We know sex has an effect of KOA. I want to know if you matched a 45 year old male to a 45 year old female, or if they also had to have the same sex.

Author’s response: Thank you for highlighting this omission. Participants were matched by sex and age which has now been included in the manuscript.

Reviewer’s comment: You state the participants were those who walk "independently," which would indicate none of them required the use of an assistive device (i.e., canes, walkers, etc.). Please clarify.

Author’s response: Thank you for highlighting this lack of clarity. The manuscript has removed independently and replaced it with; unable to stand for greater than 30 minutes or walk without gait aids/assistance

Reviewer’s comment: Lines 113-114: You state that you took the largest value and used for analysis. This was done for other dependent variables as well. I'm more familiar with using the average across three trials. As long as you can provide a reference stating this has been done prior, then I would be fine with this.

Author’s response: Maximal voluntary contraction requires the largest value be used for analysis a reference has been supplied within the manuscript.

Reviewer’s comment: Please provide reliability and validity for the system used in the gait analysis.

Author’s response: The manuscript has been edited to provide adequate reliability and validity.

Reviewer’s comment: Why were non-parametric statistical analyses performed? I would assume this was due to not meeting the assumptions for parametric analysis, which could be a result of the small sample size. Regardless, please provide details of the assumptions analysis and why you chose this method.

Author’s response: With respect to the statistical analyses, we used non-parametric analyses as we conducted a pilot study with a small sample size (Altman et al., 1983; PMID: 6405856). This has now been mentioned in the manuscript.

Reviewer’s comment: There are a lot of comparisons performed in this investigation, so setting your alpha at .05 really runs the risk of increasing your family-wise error rate. The authors should not feel pressured to "find significance" in a pilot study. Again, the main purpose will be to provide effect sizes to be used in an a priori analysis for the NEXT study and to make improvements on the methodology going forward. I would rather see you be more conservative (e.g., Bonferroni correction per analysis).

Author’s response: This is a good suggestion, a greater emphasis has now been placed on effect sizes rather than statistical inferences (e.g., comparison of p-value to an a priori set alpha level) to improve the interpretation of our results in the Discussion section. With respect to Bonferroni correction, we only compared measures between groups at one time point and thus post-hoc tests did not apply in our analyses.

RESULTS
Reviewer’s comment: Please provide another table with descriptive statistics of the participants and perform statistical comparisons between the groups (age, height, weight, etc.).

Author’s response: Thank you for highlighting this omission. The table has now been included in the manuscript.

DISCUSSION
The discussion is quite lengthy, but well-written. If the authors are struggling with a word count while trying to address any comments, I would recommend cutting this down a bit.

Reviewer’s comment: Please add a Limitations section prior to your Conclusion.

Author’s response: Limitations have now been added to the manuscript.

Reviewer’s comment: Reframe your Discussion and Conclusions per the pilot nature of this study. This is probably not a definitive investigation on this topic. Temper your conclusions appropriately. See notes above.

Author’s response: Given that this is a pilot study the hypothesis has been removed and the emphasis within the discussion and conclusion is now on the aim.

Reviewer #2: This submission examines the gaze behavior during ambulation tasks of persons with osteoarthritis of the knee and compares these behaviors (as well as some gait metrics) with age matched controls without OA of the knee.

Reviewer’s comment: The submission shares some interesting observations but it suffers from the lack of a central theory and in its current state, does not seem to present or test a clear hypothesis.

Author’s response: Given that this is a pilot study the hypothesis has been removed and the emphasis within the discussion and conclusion is now on the aim.

Reviewer’s comment: The introduction simply states that people with OA present with weakness, pain, abnormal gait and are at an increased risk for falls. It follows with a discussion of the presence of differences in gaze behaviors and balance when comparing older adults at risk for falls and younger people and finishes with a proposal that gaze behaviors in persons with knee OA have not been studied. There is no discussion of a proposed relationship between living with knee OA and the development of maladaptive gaze behaviors. In the absence of a literature on such a specific question, presenting a broader discussion of CNS adaptations to chronic orthopedic conditions could help a reader make the connection between the two phenomena being discussed. Without this grounding in theory it is difficult to see this submission as something beyond a report of two co-occurring conditions in older persons.

Author’s response: Thank you for highlighting this omission. The introduction how includes CNS adaptations to OA with a proposed link to gaze.

Reviewer’s comment: This submission presents dozens of statistical comparisons. The authors should consider focusing their paper to test a primary hypothesis and identify some key secondary hypotheses and analyses. Sticking with the current shot gun approach would require presenting corrected p-values.

Author’s response: This is a good suggestion, a greater emphasis has now been placed on effect sizes rather than statistical inferences (e.g., comparison of p-value to an a priori set alpha level) to improve the interpretation of our results in the Discussion section. With respect to Bonferroni correction, we only compared measures between groups at one time point and thus post-hoc tests did not apply in our analyses.

Reviewer’s comment: The description of the two samples needs to be more extensive. Critical information such as subjects and controls comorbidities (particularly DM), fall history, and the presence or absence of other orthopedic issues needs to be shared, as does some measure of the severity of knee OA in the OA group and the length of time they have suffered from OA.

Author’s response: This is a good suggestion for us to include in the full study. With regards to this pilot study, we have matched age, sex, heigh and weight between the groups which we believe will be a sufficient base for our full study.

Reviewer’s comment: More information is needed on the system utilized to collect gaze behaviors. How does the equipment work? Is there published data speaking to the reliability and validity of measurements collected during gait, with the system, in the studied population?

Author’s response: We have included reliability of the system to study gaze behaviors with references which includes the equipment and more information.

Reviewer’s comment: The analyses of dual task data might make more sense if the authors analyzed the differences in dual task cost as opposed to citing abnormal single task data, abnormal dual task data and proposing a possible central neural difference causing the abnormal dual task data.

Author’s response: This is a good suggestion, as a pilot study there are practical restrictions however we can certainly consider this is in the full study.

Reviewer’s comment: The FRT analysis does not fit into this paper. A static limit of stability test with the subject in a wide stance does little to inform a discussion of stability in gait, a much higher order of balance. I could make a similar argument against keeping the MVC analyses. People with KOA present with decreased force generation abilities.

Author’s response: Thank you for this suggestion. FRT has been utilized in the literature to discriminate between fallers and non-fallers which we believe may have been useful in our future study. This suggestion will be taken on, especially with regards to future research.

6. PLOS authors have the option to publish the peer review history of their article ([what does this mean?](https://journals.plos.org/plosone/s/editorial-and-peer-review-process#loc-peer-review-history)). If published, this will include your full peer review and any attached files.


If you choose “no”, your identity will remain anonymous but your review may still be made public.


**Do you want your identity to be public for this peer review?** For information about this choice, including consent withdrawal, please see our [Privacy Policy](https://www.plos.org/privacy-policy).

Reviewer #1: No

Reviewer #2: No
